# Nicotine exposure increases the complexity of dopamine neurons in the parainterfascicular nucleus (PIF) sub-region of VTA

**DOI:** 10.1186/1743-0003-11-103

**Published:** 2014-06-12

**Authors:** Die Zhang, Andrei Dragomir, Yasemin M Akay, Metin Akay

**Affiliations:** 1 Department of Biomedical Engineering, Cullen College of Engineering, University of Houston, Houston, TX 77204, USA

**Keywords:** VTA, Dopamine, Approximate entropy, Nicotine

## Abstract

**Background:**

Recent publications highlight differences within the sub-regions of the ventral tegmental area (VTA) including the parabrachial pigmented nucleus (PBP), parainterfascicular nucleus (PIF) and paranigral nucleus (PN) in the projections to the prefrontal cortex (PFC) and the glutamatergic pathway.

**Methods:**

In order to characterize the effects of prenatal nicotine exposure on the mesocorticolimbic system of the rat offspring, local field potentials were recorded from 27 sites across the VTA of 9 rats aged 40–55 days. The extracellular VTA neural activities were analyzed using Approximate Entropy (ApEn) method. Approximate entropy values were then grouped according to each anatomic location including the PBP, PIF and PN.

**Results:**

Our results have shown that the local field potentials corresponding to the neurons located in the PIF region of the VTA have ApEn values significantly higher (p = 2x10-4) in the maternal nicotine cases when compared to the saline.

**Conclusion:**

Therefore, we speculate that the dopamine neurons located in the PIF sub-region of the VTA are very likely involved with the nicotine addiction.

## Introduction

Maternal tobacco smoking during pregnancy has been established as a risk factor for the developmental and cognitive disorders in the offspring. The mesocorticolimbic system, including the ventral tegmental area (VTA), the prefrontal cortex (PFC), nucleus accumbens (NAc) and amygdala, has been implicated in motivation, learning, cognition and other reward driven behaviors. Dopaminergic (DA) neurons in the VTA play an important role in the process of these physiological functions [1-3]. Their firing patterns were shown to be important modulators for the dopamine release; thus being essential for physiological and pathological processing [4].

In one of our recent studies, we have introduced the nonlinear dynamic analysis method based on the Lempel-Ziv (LZ) method to investigate the influence of the PFC transection on the dynamics of VTA DA neural activities in the nicotine-treated rats. Our results suggested that the PFC plays a vital role in mediating VTA activity and that the LZ complexity method is a useful tool for the characterization of the dynamical changes in VTA DA neuronal firing activities [5].

Furthermore, we have extensively investigated the functional coupling between the PFC and the VTA to understand how the disruption of communication from the PFC affects the firing patterns of the VTA DA neurons. Our results suggested that exposure to nicotine triggered a significant increase in the VTA DA neuronal firing complexity when the communication between the PFC and the VTA was present, while transection obliterated the effect of nicotine. We speculate that, increased firing complexity with acute nicotine administration in the PFC-intact subjects is due to the close functional coupling between the PFC and the VTA. Furthermore, this hypothesis is supported by the fact that the deletion of the PFC results in minor alterations of VTA DA neural firing when nicotine is acutely administered.

We recently investigated the influence of nicotine exposure and prefrontal cortex (PFC) transections on VTA DA neuronal firing activities using a time–frequency method based on the continuous wavelet transform (CWT). Our results show that, systemic nicotine exposure disrupts the energy distribution in PFC-intact rats. Particularly, there is a significant increase in energy contents of the 1–1.5 Hz frequency band. These two studies led us to investigate whether or not the lack of glutamate transmission from the PFC to the VTA via in vitro exposure of VTA slices could make any change in the effects of nicotine. Our results suggested that after one hour of a single systemic nicotine injection, not all subset of synapses were potentiated.

A recent study noted that there were groups of VTA DA neurons that showed different temporal changes on the firing rate after the nicotine injection, suggesting detailed subgroups of VTA DA may exist within VTA, defined by physical function and/or anatomical sub-region [6].

In the present study, we have investigated the dynamics of the DA neurons within the VTA sub-regions in response to nicotine exposure. We hypothesize that strengthening the input from the PFC to the VTA plays an important role in the development of behavioral sensitization, which is a well-known model for addiction. To test this hypothesis, we have designed several novel electrophysiological in vitro and in vivo experiments. In this study, we characterize and quantify the dynamics of neural activity induced by gestational nicotine exposure in VTA DA neurons in three main VTA regions, including the parabrachial pigmented nucleus (PBP), parainterfascicular nucleus (PIF) and paranigral nucleus (PN) using the approximate entropy (ApEn) method. The ApEn method was chosen since it is closely related to information-theoretic methods such as entropy [7] and has been useful for the estimation of the complexity of biomedical signals with limited data samples and low signal to noise ratio [8].

## Materials and methods

All procedures were in compliance with the Guidelines for the Care and Use of Laboratory Animals. The protocol was approved by the Institutional Animal Care and Use Committee of the University of Houston.

### Animal preparations

Female Sprague–Dawley (SD) rats (Charles River), with an initial body weight of 280-300 g, were maintained on a 12 h light/12 h dark schedule at a temperature of 22 ± 2°C and 65% humidity. Access to standard food and water was unlimited. Rats were acclimated to the animal facility for 3 days before any procedures. Nicotine (Sigma), dissolved in the saline solution, was administrated to these female SD rats via osmotic mini-pumps (Alzet) with a release rate of 6 mg/kg/day. These pumps were implanted subcutaneously on the fifth day of their pregnancy and stayed there for 17 days (considering the gestational period for rats is 21 days).

### Extracellular in vivo recordings

Male offspring (40–55 days old) from those nicotine/saline treated female rats were anesthetized with chloral hydrate (400 mg/kg, i.p., with supplemental dose administrated via a lateral tail vein) and mounted in a stereotaxic apparatus (Narishige). Body temperature was maintained at 36-37°C with a homeothermic blanket system (Harvard Apparatus). The detail of extracellular recording in anesthetized rats can be found in our previous publications [1,5,6]. Briefly, spontaneous active DA cells within the VTA were determined by lowering the electrode through an area of tissue (400 × 400 μm).

9 passes separated by 200 μm were made in each rat. The sequence of the passes and anatomical boundary of the recorded area were identical among experimental subjects. Glass electrodes, filled with 2 M NaCl and 0.5% Chicago Sky Blue dye (to confirm the recording site at the end of each experiment using standard histological procedures) [1], with final resistance ranging from 6 to 8 MΩ were used in this study. The recording electrode was placed into the VTA through a small burr hole on the skull (about 3.0 mm anterior to the lambda and 0.9 mm lateral to the midline) by a motor-step micromanipulator (Narishige), with the reference attached scalp. DA neurons were usually found between 7.0 and 8.5 mm below the cortical surface, and identified according to the criteria well described in previous studies [1,5,6]: biphasic (positive–negative) or triphasic (positive–negative-positive) spike waveform with a duration greater than 3.0 ms, a broad initial positive phase (>1.1 ms, measured from the start of action potential to the negative trough), an initial segment-soma to dendritic break in the initial positive phase, and a slow firing rate (<10 spikes/s). Single unit signals and LFPs were recorded simultaneously with same glass electrode and without hardware filtering. All raw data, including single-unit firing and local field potential signals, were collected on-line via multi-channel recording system (Plexon) to the work station computer. During the pre-processing, LFPs were low-pass filtered with a 5^th^ order Butterworth filter with 200Hz cut-off and subsequently down-sampled at 1 kHz. As noted by previous studies, a common approach for removing the LFP (and other neural signals) contamination with spikes as well as artifacts from other physiological sources is the wavelet-based denoising [9-12]. The goal is to transform the noisy data into an orthogonal representation in time-frequency domain. Thresholding is then applied to the wavelet coefficients thus removing the noise, and the modified coefficients are used to reconstruct the original signal in time domain. We used a Daubechies 4 wavelet and decomposed our LFP signals into 8 levels. The highest two levels’ detail coefficients and the approximation coefficients were used to reconstruct the denoised signals, the rest being set to zero.

### Approximate entropy (ApEn) method

The approximate entropy method (ApEn) is a nonlinear measure quantifying the regularity of a time-series, which is particularly efficient in the case of short and noisy data sets and can be employed in the analysis of both stochastic and deterministic signals [7,8]. This feature is crucial in the case of biosignals, which are outputs of complex biological networks and may be deterministic, stochastic or both. ApEn provides a model-independent measure of the irregularity of the signals. The algorithm characterizes a time series into a non- negative number, with higher values representing more complex systems. Basically, the ApEn measures the logarithmic likelihood that runs of temporal patterns that are close within a certain threshold *r* over a defined number of observations *m* remain close in the next incremental comparisons. A higher likelihood for these segments of remaining close within the defined threshold produces smaller ApEn values, indicating a higher level of regularity.

In this study, we used the ApEn to quantify the rate of new pattern generation along given sequences of symbols as a measure of the complexity (regularity) of DA neural firing. The DA neural activity arises from complex feedback networks and non-linear interconnections between VTA DA neurons and neurons from other brain areas including the PFC, NAc, and amygdala. Therefore, the neural activities are inherently complex (irregular) and the ApEn could be an appropriate method to the underlying nonlinear dynamics of the DA neural firing.

The ApEn complexity has been applied extensively in biomedical signal analysis as a metric to estimate the complexity of discrete-time physiologic signals, proving its robustness over other complexity/entropy measures.

In the present study, we used m = 2 and r = 0.1·SD(*x*(*i*)), where SD(*x*(*i*)) represents the standard deviation of the LFP signal. More details on the ApEn method and its implementation are given elsewhere [7,13].

## Results

**Figure 1:**
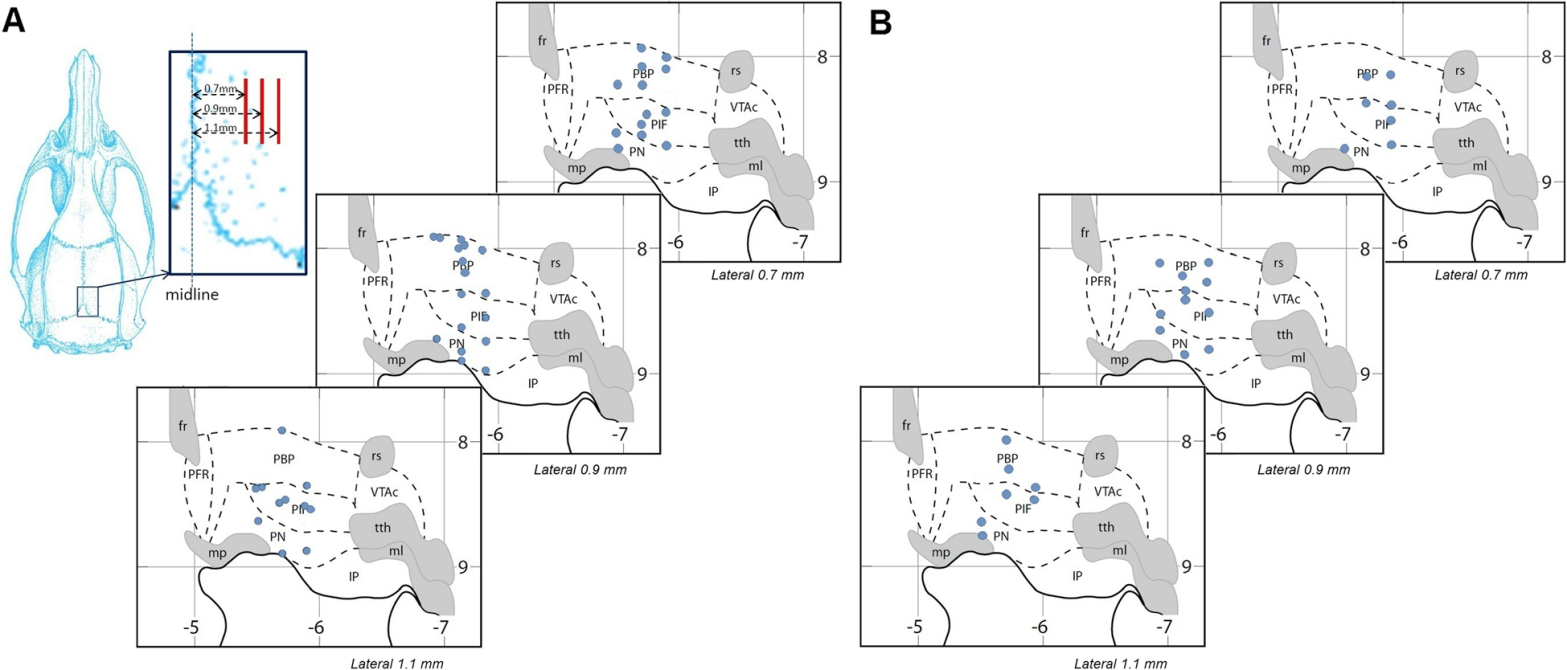
**Local field potential recording sites in VTA.** LFPs were recorded on: **(A)** 41 VTA DA neurons from offspring of dams exposed to nicotine during gestational period, and **(B)** 25 from offspring of saline treated dams as control.

**Figure 2:**
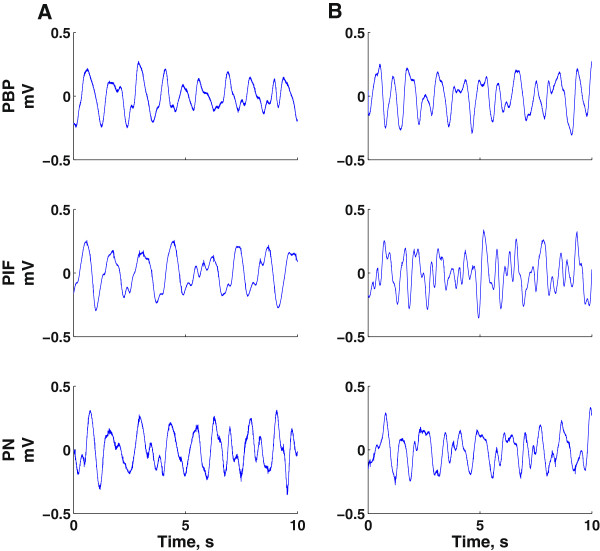
**Typical LFP traces recorded from VTA.** This figure shows the typical LFP traces from the PBP, PIF and PN. Left panels **(A)** is of control group rats, and right panels **(B)** is of rats from the maternal nicotine exposure group.

A total of 9 offspring rats were used in the present study, 6 from dams exposed to nicotine during gestational period, while the remaining three were from dams which received the saline treatment, as described in the Methods section. Before analysis of the LFP recordings using the ApEn method, all data samples were denoised using the wavelet transform and detrended by removing their mean. Recordings were performed from several sites across the VTA, targeting three major sub-regions, namely the PBP, PIF and PN, as shown in Figure 1A and B. Generally we recorded ~3 min of LFP activity from each recording site. For each individual recording a number of six consecutive 10s long segments were chosen for ApEn analysis, after ~1 minute of stable recording was achieved. A total of 66 recordings were performed from 27 different sites within the VTA, 25 from offspring of saline treated dams (control group) and 41 from offspring of dams exposed to nicotine during gestational period (maternal nicotine exposure group).Figure 2 shows typical LFP traces recorded from the different VTA sub-regions of control group rats (left panels) and of rats from the maternal nicotine exposure group (right panels). The top panels display LFPs recorded from the PBP sub-region, the middle panels show LFP signals from the PIF sub-region, while the bottom panels display signals recorded from the PN sub-region and for all recordings within a specific VTA sub-region (PBP, PIF and PN) pertaining to the specific animal.

**Figure 3:**
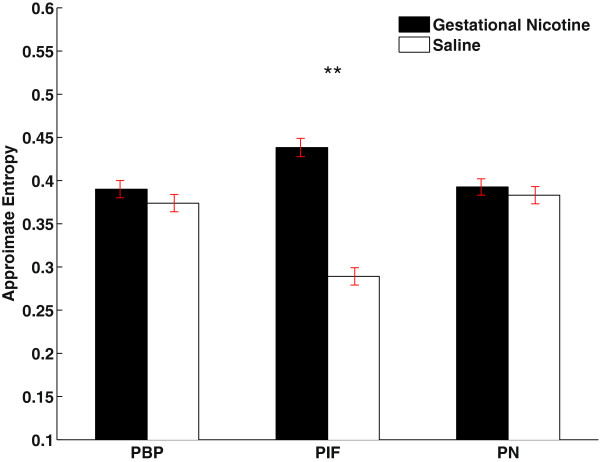
**Gestational nicotine significantly increased the approximate entropy values of LFPs in PIF.** This bar plot shows the alterations on approximate entropy values between gestational nicotine treated group and control group in PBP, PIF and PN. The difference in the case of the PIF is statistically significant when comparing between control and Gestational nicotine exposure.

We generally observed slower oscillatory rhythms within the PIF sub-region of the control group (saline-treated dams), while faster oscillations were observed in the PIF of maternal nicotine exposure group. The slower, rhythmic oscillations are usually a hallmark of synchronous neural activity of the neurons, while the faster and generally smaller amplitude oscillations in the latter case may be an indicator of less synchronized, more random neural activity in the respective area. Throughout the recordings most of the power was found in the [0–3] Hz frequency band, consistent with the observations of previous studies.Figure 3 summarizes the results for all rats under study. The mean approximate entropy values estimated from LFPs recorded within the PBP sub-region were 0.37 ± 0.009 (SEM) and 0.39 ± 0.005 for the rats in control group and maternal nicotine exposure group, respectively. The largest differences were observed for the PIF sub-region, where the mean values were 0.28 ± 0.007 (control) and 0.43 ± 0.01 (maternal nicotine exposure), while in the case of the PN the mean approximate entropy values were 0.38 ± 0.01 (control) and 0.39 ± 0.009 (maternal nicotine exposure).

Statistical analysis using the Student’s t-test revealed that the difference in the case of the PIF is statistically significant when comparing between control and maternal nicotine exposure (p < 10^-4^), while in the case of PBP and PN the differences were found not significant (p > 0.1).

## Discussion

Nicotine, the biologically active substance of tobacco, is reported to increase VTA DA neuronal firing rate, bursting and enhance dopamine release. A number of studies have previously indicated that gestational nicotine exposure induces complex alterations in the development of the offspring brain, including a multitude of neurochemical changes (such as reduced DNA synthesis), as well as neurotransmitter dysfunction [14] and decreased numbers of dopaminergic neurons within the VTA [15]. However, because of the anatomical and functional differences in different areas in the brain and the complex neural networks in the VTA, the mechanism by which nicotine affects VTA DA neurons firing is still not clear.

Sub-regions within the VTA are not necessarily consistent across different studies, especially when different species are involved [16-20]. The present studies generally divided the VTA into four major zones: PN, PBP, parafasciculus retroflexus area (PRF), and ventral tegmental tail (VTT). However, only the PN and PBP are dopaminergic cell-body-rich zones. PIF is also suggested to be included in the dopamine system because of its critical contribution to the dopamine reward circuitry and the heavy dopaminergic inputs to the ventromedial striatum [21]. It is well known that the subgroups of the VTA dopamine neurons have different projection targets. It seems likely that this will relate to the functionally distinct populations recorded. Our recent study suggested that there were groups of VTA DA neurons that showed different responses to the nicotine, confirming the subgroups of VTA DA may exist, not only defined by anatomical sub-regions, but also by their physiological functions [6].

This study aims to shed light on the critical alterations of the neural activity within the VTA of rats emerging from maternal exposure to nicotine. We recorded local field potentials from several sites within the VTA of the rat brain and compared the complexity of the signals recorded from control rats with that of the rats from dams that were exposed to nicotine during gestation. The recorded field potential signals from the VTA were analyzed using the approximate entropy (ApEn). The ApEn method was chosen since it is a nonlinear analysis method commonly used to assess irregularity (complexity) of biomedical signals [22,23]. It is widely reported that, the quantitative changes in the complexity of electrophysiological signals can be accurately assessed using nonlinear dynamics analysis methods [24]. Generally, brain electrophysiological signals are thought to originate from complex nonlinear systems [25]. Since local field potentials reflect the sum of the postsynaptic potentials of the groups of local cells, they reflect the integrated properties of the local neural activity and such a system has complex, dynamic behavior. Therefore, nonlinear dynamics methods, such as the ApEn, have been widely used for the analysis of the electrophysiological signals.

Traditionally, VTA DA neuronal firing irregularity has been assessed with various methods based on inter-spike intervals (ISI) distribution of the neuronal spike trains. While conventional methods such as coefficient of variation (CV) or firing rate statistics [1,3,26] may provide important insights, they fail to appropriately account the underlying neural dynamics. To this goal, previous studies used the nonlinear prediction method to quantify the chaotic behavior of VTA DA neuronal ISIs in aging-related studies, and assessed nonlinear deterministic structures in ISI firing patterns, respectively [27,28]. Other studies focused on LFPs recorded from VTA and used frequency domain analysis to investigate the low frequency oscillations of dopamine neural activity and an autocorrelation method to identify repeating patterns in the time domain signals with the goal of assessing the attenuations in depressive-like behavior after deep brain stimulation [29].

## Conclusion

We demonstrated that maternal nicotine exposure influences the complexity of the DA neurons in the VTA of the offspring brain in a non- uniform manner, with the most affected area being the PIF. This observation reinforces observations from the recent studies, which suggested significant differences (both functional and anatomical) between different regions [30] and dopaminergic neurons within the VTA [31].

We speculate that the significantly lower complexity values observed in the PIF sub-region of the VTA from the control group result from the synchronous activity of a highly homogeneous group of neurons. The higher complexity observed in the PIF sub-region of the maternal nicotine exposure group may be the outcome of a more random neural activity resulting from a population of neurons with decreased homogeneity.

## Competing interests

The authors declare that they have no competing interests.

## Authors’ contributions

The authors contributed equally to this work. DZ and YMA contributed to the design and implementation of experiments. AD and MA contributed to data analysis. MA contributed in the supervision of the research study. All authors read and approved the final manuscript.
